# Impaired Brain Energy Metabolism: Involvement in Depression and Hypothyroidism

**DOI:** 10.3389/fnins.2020.586939

**Published:** 2020-12-04

**Authors:** Katarzyna Głombik, Jan Detka, Anna Kurek, Bogusława Budziszewska

**Affiliations:** Laboratory of Immunoendocrinology, Department of Experimental Neuroendocrinology, Maj Institute of Pharmacology, Polish Academy of Sciences, Kraków, Poland

**Keywords:** depression, hypothyroidism, brain, metabolism, animal disease model

## Abstract

Although hypothyroidism appears to be an important factor in the pathogenesis of depression, the impact of thyroid hormones on the bioenergetics of the adult brain is still poorly known. Since metabolic changes are reported to be a key player in the manifestation of depressive disorder, we investigated whether there are differences in selected metabolic markers in the frontal cortex and hippocampus of Wistar Kyoto rats (WKY; an animal model of depression) compared to those of control Wistar rats and whether the induction of hypothyroidism by propylthiouracil (PTU) elicits similar effects in these animals or intensifies some parameters in the WKY rats. In our study, we used WKY rats as a model of depression since this strain exhibits lower levels of monoamines in the brain than control rats and exhibits behavioral and hormonal alterations resembling those of depression, including increased reactivity to stress. The findings indicate a decrease in glycolysis intensity in both brain structures in the WKY rats as well as in both strains under hypothyroidism conditions. Furthermore, hypothyroidism disrupted the connection between glycolysis and the Krebs cycle in the frontal cortex and hippocampus in the depression model used in this study. Decreased thyroid hormone action was also shown to attenuate oxidative phosphorylation, and this change was greater in the WKY rats. Our results suggest that both the depression and hypothyroidism models are characterized by similar impairments in brain energy metabolism and mitochondrial function and, additionally, that the co-occurrence of hypothyroidism and depression may exacerbate some of the metabolic changes observed in depression.

## Introduction

Depression affects more people than all other mental illnesses combined, and the currently available pharmacotherapies and behavioral therapies are not effective in many patients. The low efficacy of pharmacotherapy is probably because the antidepressant drugs currently in use mainly affect noradrenergic and serotonergic neurotransmission, while many studies have shown that this disease involves not only disturbances in neurotransmission but also changes in hormonal systems, immune systems, and energy processes that together impair synaptic plasticity, leading to numerous functional changes, including depressed mood (Horowitz and Zunszain, 2015). The participation of thyroid hormones in the pathogenesis of depression is evidenced by epidemiologic data that indicate that thyroid dysfunction often leads to the development of mental diseases; among patients with affective disorders, 1–4% have hypothyroidism, and 4–40% present subclinical hypothyroidism (Duntas and Maillis, 2013; Loh et al., 2019). The therapeutic effectiveness of adjunctive treatment of refractory depression with superphysiological doses of thyroid hormones is well established, but the mechanism of action of thyroid hormones on the function of the central nervous system in adults is not well known (Bauer et al., 2002, 2016). The lack of such research mainly results from the fact that although thyroid hormones regulate many important processes during development, such as growth, differentiation, migration, neuronal integration, glial cell proliferation, myelination and neurotransmitter synthesis, for a long time, it was thought that after development, the brain was no longer a target tissue for the action of these hormones. Therefore, although the relationship of thyroid dysfunction with mood disorders and cognitive function has been observed clinically for a long time, the effects of thyroid hormones on processes occurring in the brain after the developmental period are unclear. Understanding the role of thyroid hormones in the pathogenesis of depression is also hampered by the fact that pro-depressive effects are observed not only in hypothyroidism but also sometimes in hyperthyroidism and because these hormones can, in addition to exerting genomic effects, make an impact on the membranes of brain cells.

Although the vast majority of people with depression do not exhibit hypothyroidism, abnormal T4 to T3 ratios, elevated reverse T3 (rT3) levels, blunted TSH responses to TRH, and the presence of antithyroid antibodies are reported to be more common in people with depression than in the general population (Hage and Azar, 2012; Pilhatsch et al., 2014). Moreover, some studies suggest that depression is connected with a decrease in the level or activity of thyroid hormones only within the brain. This is likely because the concentration of active thyroid hormone T3 in the brain depends not only on its synthesis in the thyroid gland but also, to a large extent, on the expression of transporters and deiodinase enzymes in particular brain structures (Flamant et al., 2015). Moreover, thyroid hormone action also depends on the expression and sensitivity of thyroid hormone nuclear receptors, so thyroid hormone signaling in specific brain cells may be changed in depression. The concept that tissue-specific changes in thyroid hormone action may occur in depression even with normal thyroid hormone concentrations in the blood and pituitary gland was confirmed by data, indicating that treatment with high doses of thyroid hormones is well tolerated in patients with depression but not in healthy people (Kelly and Lieberman, 2009; Kelly, 2016).

Current research indicates that metabolic disturbances in the brain that lead to dysfunctional neurotransmission, behavior, and cognition, play a significant role in the pathogenesis of depression. Since it has been found that changes in glucose metabolism occurring in depression are normalized by clinically effective adjunctive therapy with levothyroxine (L-T4) or T3, it is therefore possible that one of the causes of depression may be a weakening of the metabolic effects of thyroid hormones in the brain (Iosifescu et al., 2008; Bauer et al., 2010, 2016).

The aim of the present study was to demonstrate the following: (1) whether there are changes in the parameters characteristic of brain energy metabolism and mitochondrial function in an animal model of depression (Wistar-Kyoto rats); (2) whether hypothyroidism (PTU administration to Wistar rats) causes alterations in mitochondrial metabolic markers similar to those observed in the depression model and which metabolic markers are changed in each condition; and (3) whether changes in some stages of metabolism are intensified in the model of comorbidity of depression and hypothyroidism (Wistar-Kyoto rats + PTU) compared to those in models 1 and 2. In these studies, we used the Wistar-Kyoto (WKY) rats, a strain that exhibits changes in neurotransmitter levels and hormonal and behavioral disturbances resembling those in depression, as a model of depression. Existing studies have shown that WKY rats have lower levels of monoamines (5-HT, NE, and DA) in several brain structures, elevated levels of glutamate in the prefrontal cortex, and reduced concentrations of BDNF in the plasma (Aleksandrova et al., 2019). Moreover, WKY rats exhibit hormonal alterations characteristic of depression, including dysregulation of the hypothalamic–pituitary–adrenal (HPA) and hypothalamic–pituitary–thyroid (HPT) axes. Data concerning WKY rats demonstrated an increase in the mRNA expression levels of CRF receptors, ACTH, and POMC in the anterior pituitary and enlarged adrenal glands. WKY rats were also characterized as a strain with increased reactivity to stress, prolonged ACTH and corticosterone secretion, and weakened negative feedback mechanisms (Aleksandrova et al., 2019). In contrast to the HPA axis, few data are available on HPT axis disturbances in these rats; however, higher plasma concentrations of T3 and TSH were found in the WKY rats than in the Wistar rats (Solberg et al., 2001). A simultaneous increase in TSH and T3 in some hours of the diurnal cycle suggests a disturbance in the mechanism of HPT axis regulation and resembles the subclinical hypothyroidism common in depressed patients (Solberg et al., 2001). Consistent with the biochemical disturbances, the behavioral changes also observed in the WKY rats, such as hypolocomotion, increased immobility in the forced swim test, disruptions in the sucrose preference test, enhanced response to stress, and impaired learning and memory processes, resemble changes that occur in depression. For these reasons, this strain, which is endogenously susceptible to stress, is an established genetic model of depression and is the most suitable control group in the context of depression studies in Wistar rats. It has been shown that animals of these strains have common genetic background but differ in their susceptibility to stress (Tizabi et al., 2012; Nam et al., 2014). Additionally, since the WKY rats are partially resistant to antidepressant drugs, these rats are considered to be a model of treatment-resistant depression. Interestingly, in this disease, adjunctive treatment with superphysiological doses of thyroid hormones exhibits clinical efficacy (Lahmame et al., 1997; Redei et al., 2001; Solberg et al., 2001; Willner and Belzung, 2015; Willner et al., 2019). The frontal cortex and hippocampus were selected for current study because they are the main structures affected in depressive disorders. As indicated by recent studies, including our research conducted in a prenatal stress animal model of depression, metabolic changes may participate in the pathogenesis of depression (Rezin et al., 2008; Regenold et al., 2012; Detka et al., 2015; Głombik et al., 2017). The metabolic action of thyroid hormones in peripheral tissues and in the developing brain is well documented, and a few studies have indicated that these hormones increase the activity of Krebs cycle enzymes and affect mitochondrial respiration in the adult brain (Katyare et al., 1994; Bauer et al., 2016). For this reason, we compared the selected metabolic parameters in the WKY rats and the control Wistar rats under basal conditions and under conditions of decreased thyroid hormone synthesis (obtained by the administration of a thyroid peroxidase inhibitor). We examined markers of glycolysis, the Krebs cycle, and oxidative phosphorylation that were selected mainly on the basis of the results of our previous studies conducted in a prenatal stress animal model of depression (Detka et al., 2015; Głombik et al., 2018, 2020). Since disturbances in mitochondrial respiration were demonstrated in both depression and hypothyroidism in the present study, the expression of oxidative phosphorylation complexes and mitochondrial activity in the various stages of respiration were analyzed in mitochondrial fractions isolated from the frontal cortex and hippocampus.

To show whether there are changes in factors regulating thyroid hormone levels or action in the brain in the models studied, we measured the expression of type 2 deiodinase (DIO2), type 3 deiodinase 3 (DIO3), thyroid receptors (TRα1 and TRβ1) and retinoid receptors (RXRα and RXRβ) in the frontal cortex and hippocampus. In the brain, DIO2 is predominantly expressed in glia and is responsible for the transformation of T4 to active T3 that is then delivered to neurons, whereas DIO3 is mainly involved in the inactivation of thyroid hormones (Morte and Bernal, 2014). The biological activity of T3 is mainly mediated by thyroid hormone nuclear receptors (TRs), which are transcription factors that bind to thyroid hormone response elements (TREs) in the regulatory regions of target genes. Although TRs are capable of binding to TREs without auxiliary factors, they preferentially bind to many TREs as heterodimers with retinoid X receptors (RXRs). To determine whether there are changes in the particular receptor isoforms present in the studied models, the expression levels of TRα1 (the predominant isoform in the central nervous system), TRβ1, and the retinoid X receptors RXRα and RXRβ, which often form heterodimeric complexes with TRs, were assayed.

## Materials and Methods

### Animals and Treatments

All experiments were performed according to the National Institutes of Health Guide for the Care and Use of Laboratory Animals and were approved by the Local Ethics Committee in Kraków, Poland (permission no. 46/2018 of 01.02.2018).

Eight-week-old Wistar and WKY male rats (Charles River Laboratories, Hamburg, Germany) were maintained at a room temperature of 22 ± 2°C on a 12-h light/dark cycle (lights on at 6:00 am) with food available *ad libitum*. Animals were randomly assigned to four groups, each containing 10 animals: group I, control, Wistar rats fed a standard diet and drinking water *ad libitum*; group II, endogenous depression, WKY rats fed a standard diet and drinking water *ad libitum*; group III, hypothyroid, Wistar rats fed a standard diet and treated with 0.05% (w/v) propylthiouracil (PTU) in drinking water for 3 weeks; and group IV, coexistent depression and hypothyroidism, WKY rats fed a standard diet treated with 0.05% (w/v) PTU in drinking water for 3 weeks. PTU is an antithyroid drug which inhibits synthesis of thyroid hormones, used in the therapy of hyperthyroidism and Graves disease.

### Body Weight Gain Measurement

Body weight gains (g) were measured once a week.

### Forced Swim Test (FST)

The FST, one of the most commonly used assays, is a scoring technique wherein immobility, swimming, and climbing behavior are distinguished to measure depression-like behaviors in rodents and to predict antidepressant drug effects (Yankelevitch-Yahav et al., 2015). The FST was conducted in accordance with the procedure described by Porsolt et al. (1978). Animals (*n* = 10 per group) were forced to swim in a cylinder filled with water to a height of 35 cm for 5 min, during which the total immobility time and climbing time were measured. The actual test in animals was preceded by a pretest carried out on the previous day during which the animals were placed in the water-filled cylinder for 15 min.

### Novel Object Recognition (NOR) Test

The NOR test is a memory test that estimates the ability of rodents to recognize a novel object in the environment and is designed to assess recognition memory (Antunes and Biala, 2012). The NOR test (Taglialatela et al., 2009) was applied with modifications. This test is based on the spontaneous tendency of rats to spend more time exploring a novel object than a familiar object. Twenty-four hours after a habituation trial, during which the rats were allowed to explore an empty container, the animals were placed in the same area with two identical objects set at an equal distance for 3 min. After 1 h, the rats were placed in the same container in the presence of the familiar object and a novel object similar in size and height but different in shape and appearance. The time spent by every animal exploring each object was measured. The results are presented as the preference index, which was calculated as follows: preference index = (time with novel object/[time with novel object + time with familiar object]).

### Biochemical Analysis

All biochemical experiments were carried out under exactly the same conditions for every sample, regardless of the type of animal treatment.

The rats were sacrificed under non-stressful conditions (between 9:00 a.m. and 12:00 a.m.) by rapid decapitation, after which trunk blood samples were collected into plastic test tubes containing EDTA (KABE LABORTECHNIK, Nümbrecht, Germany). After centrifugation (3,000 rpm, 20 min, 4°C), the separated plasma was collected and frozen at −20°C. The brains were removed, and the frontal cortex and hippocampus were rapidly dissected on ice-cold glass plates, frozen on dry ice and stored at −80°C, excluding the samples collected for mitochondrial respiratory experiments (Oxygraph-2K study), in which freshly isolated brain tissues were used (as described later).

### Thyroid Hormone Measurement in the Plasma and in the Brain

The plasma concentrations of TSH (Demeditec, Kiel, Germany), free T3 and free T4 (both: DiaMetra, Perugia, Spello, Italy) were assayed using ELISA according to the instructions provided by the manufacturer. The concentrations of TSH, free T3, and free T4 in each sample were calculated from the standard curve and are displayed as ng/ml, pg/ml, and ng/dl, respectively. The T3 level in the frontal cortex and hippocampus was measured using the ELISA assay described above.

### Total Cholesterol, HDL, LDL, Triglyceride, and Glucose Measurement in the Plasma

Biochemical analyses of plasma glucose and lipid profiles were performed on a Mindray BS-800 Chemistry Analyzer (Mindray, Shenzhen, Guangdong, China). The concentrations of the measured factors are displayed as mg/dl.

### Corticosterone Measurement in the Plasma

The corticosterone concentrations were measured by using an ELISA method with a commercially available assay kit (Fine Test, Wuhan, China) and are displayed as ng/ml.

### Gene Expression Study

Total RNA was extracted from the frontal cortex and hippocampus using a Total RNA Mini kit (A&A Biotechnology, Gdynia, Poland), after which the RNA concentrations in the samples were measured using a NanoDrop ND-1000 spectrophotometer (Thermo Fisher Scientific, Waltham, MA, United States). Conversion into cDNA was performed by a High-Capacity cDNA Reverse Transcription Kit (Thermo Fisher Scientific, Waltham, MA, United States) using a T100 Thermal Cycler (Bio-Rad, Hercules, CA, United States). Quantitative real-time PCR was performed using TaqMan probes and primers for the *thra*, *thrb*, *rxra*, *rxrb*, *dio2*, *dio3*, and *thrsp* genes (Thermo Fisher Scientific, Waltham, MA, United States) and the FastStart Universal Probe Master (Rox) kit (Roche, Basel, Switzerland) using the CFX96 Real-Time System (Bio-Rad, Hercules, CA, United States). The following thermal cycling conditions were used: 2 min at 50°C, 10 min at 95°C, followed by 40 cycles of 95°C for 15 s and 60°C for 1 min. The *C*t values for each sample were measured in the exponential phase of the PCR, and the ΔΔ*C*t method was used for data analysis. *hprt1* (Thermo Fisher Scientific, Waltham, MA, United States) was used as the reference gene.

### Isolation of the Mitochondria-Enriched Membrane Fraction and Cytosolic Fraction

To measure the activities and amounts of selected mitochondrial enzymes, mitochondria-enriched membrane fractions were isolated from the frontal cortex and hippocampus by using the procedure described by Wernicke et al. (2010). Briefly, brain tissues were homogenized in ice-cold homogenization buffer [containing 5 mol/l HEPES/NaOH, pH 7.4, 320 mmol/L sucrose, and 1 mmol/L Na+/EDTA with the addition of 0.5% protease inhibitor cocktail (Thermo Fisher Scientific, Waltham, MA, United States)] using a motor-driven Teflon-glass homogenizer and centrifuged at 1,300 × *g* for 4 min at 4°C. After the supernatants were collected, the remaining pellet was rinsed twice with homogenization buffer and centrifuged at 1,500 × *g* for 4 min at 4°C to increase mitochondrial yield. The combined supernatants were then centrifuged at 17,000 × *g* for 12 min at 4°C, after which the mitochondria-containing pellets and supernatants (cytosolic fraction) were stored at −80°C.

### Western Blotting to Detect OXPHOS, UCP4, Mitofusin 2, VDAC1, and HK1

Samples of isolated mitochondria-enriched membrane fractions for Western Blotting were prepared through lysis in PBS containing 1% Triton X-100 and 0.1% SDS with phosphate and protease inhibitors (Thermo Fisher Scientific, Waltham, MA, United States). SDS-PAGE was performed under a constant voltage of 150 V. Proteins were transferred to PVDF membranes at a constant current of 150 mAmp for 2 h in CAPS buffer. Some membranes were cut to allow simultaneous incubation with different antibodies. After 1 h of blocking in 5% non-fat milk [in PBST with 0.05% Tween 20 (Sigma-Aldrich, Saint Louis, MO, United States)], membranes were incubated overnight with Total OXPHOS Rodent WB Antibody Cocktail (6.0 μg/mL, Abcam, Cambridge, United Kingdom) and UCP4 polyclonal (1.0 μg/mL, Thermo Fisher Scientific, Waltham, MA, United States), Mitofusin 2 monoclonal (1.0 μg/mL, Abcam, Cambridge, United Kingdom), VDAC1 monoclonal (1.0 μg/mL, Abcam, Cambridge, United Kingdom), or HK1 polyclonal (1:1000, Proteintech, Wuhan, China) antibodies diluted using a SignalBoost Immunoreaction Enhancer Kit (Merck, Darmstadt, Germany). The next day, after rinsing 3 × 10 min with PBS with 0.05% Tween 20, the blots were incubated for 1 h at RT with either a horse anti-mouse or goat anti-rabbit IgG HRP-conjugated secondary antibody (Vector Laboratories, Peterborough, United Kingdom), washed 4 × 10 min in TBST (Tris-buffered saline with 0.05% Tween 20), and developed using the BM Chemiluminescence Western Blotting Substrate (POD) (Roche, Mannheim, Germany). Chemiluminescence was visualized with a luminescence image analyzer (Fujifilm LAS-1000 System) and quantified using Fujifilm Multi Gauge software (Fujifilm, Tokyo, Japan). For some blots, antibody stripping was performed by incubation in 100 mL of Tris–HCl (pH 6.7) containing 2% SDS and 700 μL of 2-mercaptoethanol (all from Sigma-Aldrich, Saint Louis, MO, United States). Then, the membranes were washed 3 × 10 min in TBST, blocked, and reprobed with an antibody against β-actin (1: 15,000; Sigma-Aldrich, Saint Louis, MO, United States) diluted using a SignalBoost Enhancer Kit as an internal loading control (Merck, Darmstadt, Germany). The intensity of each target protein band was divided by the intensity of the internal loading control (β-actin) for that sample to adjust the target protein signals with respect to small, unavoidable variations in sample loading. The ratio of the intensity of the target protein band to that of β-actin was used to compare target protein abundance in different samples.

### L-Lactate Estimation

The level of lactate in the samples was measured with a colorimetric assay kit (BioVision, Milpitas, CA, United States). The mitochondria-enriched membrane fraction and cytosolic fraction were transferred to a 96-well plate and mixed with the reaction mix. After a 30-min incubation, the absorbance was measured at a wavelength of λ = 570 nm using a spectrophotometer (Tecan Infinite M200 Pro, Männedorf, Switzerland). The concentration of lactate in each sample was calculated from the standard curve and is displayed as nmol/mg protein.

### Pyruvate Estimation

A fluorimetric assay kit (BioVision, Milpitas, CA, United States) was used to measure the concentration of pyruvate in the cytosolic fraction isolated from the frontal cortex and hippocampus. Samples and pyruvate standards were incubated in a 96-well plate with 50 μl/well of reaction mix for 30 min, after which the fluorescence was measured at Ex/Em = 535/590 in a fluorometer (Tecan Infinite M 1000, Männedorf, Switzerland). The concentration of pyruvate was then calculated from the standard curve and is displayed as nmol/mg protein.

### Pyruvate Dehydrogenase, Pyruvate Dehydrogenase Kinase Isozyme 2 (PDK2), and Pyruvate Dehydrogenase Kinase Isozyme 4 (PDK4)

The concentrations of pyruvate dehydrogenase, PDK2, and PDK4 in the mitochondria-enriched fractions of the frontal cortex and hippocampus were determined using an ELISA method (PDH: EIAab Science Co., Wuhan, China; PDK2 and PDK4: Wuxi Donglin Sci & Tech Development Co., Wuxi, China) according to the manufacturers’ instructions. Samples and standards were dispensed in precoated 96-well ELISA plates and incubated. The absorbance was measured using an Infinite M200 Pro spectrophotometer (Tecan, Männedorf, Switzerland) at a wavelength of λ = 450. The concentrations of the examined proteins in the samples were calculated from a standard curve and are expressed as ng/mg protein for pyruvate dehydrogenase and as pg/mg protein for PDK2 and PDK4.

### Aconitase Activity

Aconitase activity was measured in mitochondria-enriched fractions derived from both examined brain structures using an Aconitase Activity Colorimetric Assay Kit (BioVision, Milpitas, CA, United States) according to the manufacturer’s instructions. The samples were sonicated, activated with Aconitase Activation Solution containing equal amounts of cysteine-HCl and (NH_4_)_2_Fe(SO_4_)_2_, and subsequently transferred to 96-well plates along with standards (0, 4, 8, 12, 16, and 20 nmol/well). A total of 50 μL/well of reaction mix (42 μL of assay buffer, 2 μL of substrate, and 6 μL of enzyme mix) was added to each test sample and standard. After a 45-min incubation period and the addition of 10 μL of Developer to each well, the absorbance was measured at a wavelength of λ = 450 nm using a spectrophotometer (Tecan Infinite M200 Pro, Männedorf, Switzerland). Aconitase activity was calculated from a standard curve and is expressed as mU/mg protein.

### Mitochondrial Respiratory Function

Mitochondrial respiration was measured at 37°C by high-resolution respirometry with an Oxygraph-2k (Oroboros Instruments, Innsbruck, Austria) as described (Pesta and Gnaiger, 2012; Sebastian et al., 2012). Frontal cortices and hippocampi isolated from rats were homogenized in ice-cold homogenization buffer [0.25 M sucrose, 50 mM KCl, 5 mM EDTA, 1 mM sodium pyrophosphate, 5 mM MgCl_2_ (pH 7.4), Sigma-Aldrich, Saint Louis, MO, United States] with the addition of protease inhibitor cocktail (Thermo Fisher Scientific, Waltham, MA, United States) using a rotary Teflon-glass homogenizer. Tissue homogenates were centrifuged at 1,300 rpm for 10 min at 4°C. After the supernatant (SN1) was collected, the remaining pellet was resuspended in 4 volumes of homogenization buffer and centrifuged under the same conditions to maximize mitochondrial yield. The obtained supernatant (SN2) was then mixed with SN1, and the supernatant mixture was centrifuged at 9,000 × *g* for 15 min. After gentle resuspension of the pellet containing the mitochondria, the protein concentration was measured using the BCA method (Smith et al., 1985). Three hundred micrograms of isolated mitochondria suspended in 2 mL of MiR05 respiration buffer (0.5 mmol/L EGTA, 3 mmol/L MgCl_2_.6H_2_O, 60 mmol/L *K*-lactobionate, 20 mmol/L taurine, 10 mmol/L KH_2_PO_4_, 20 mmol/L HEPES, 110 mmol/L sucrose, and 1 g/L fatty acid-free BSA, pH 7.0 with KOH, Sigma-Aldrich, Saint Louis, MO, United States) per Oxygraph-2k chamber was used for each experiment. All respiration measurements were made with the following protocol: glutamate (10 mM) and malate (2 mM) without ADP [L: LEAK (CI)]; respiration assessed by the addition of 2.5 mM ADP [P: OXPHOS (CI) capacity state]; and then the addition of 10 mM succinate [E: ETS (CI + II)]. Then, the uncoupling control was measured by the titration of the protonophore carbonylcyanide-4-(trifluoromethoxy)-phenylhydrazone (FCCP) (FCCP-uncoupled state, max ETS) (Pesta and Gnaiger, 2012; Sebastian et al., 2012). Finally, rotenone (1 μM, E: ETS (CII) state) and antimycin A (2.5 μM) were added to inhibit complex I and complex II, respectively. The inhibition of respiration in uncoupled mitochondria allows for the evaluation of oxygen flux due to oxidative side reactions (residual oxygen consumption, ROX). Data are presented as % of control of respiratory flux control ratios, corrected with residual oxygen consumption (ROX) and normalized to max ETS (after FCCP titrations). The ETS-coupling efficiency and OXPHOS-coupling efficiency were calculated based on the obtained data according to the following equations: ETS-coupling efficiency = (ETS capacity − LEAK respiration)/ETS; OXPHOS-coupling efficiency = (OXPHOS capacity − LEAK respiration)/OXPHOS.

### Protein Measurement

The protein concentration in tissue homogenates was measured with the bicinchoninic acid method (Smith et al., 1985) using a Pierce^TM^ BCA Protein Assay Kit (Thermo Fisher Scientific, Waltham, MA, United States).

### Statistics

Statistical evaluations were performed using the Statistica 13.3 software and consisted of two-way analyses of variance (ANOVA) followed by the Duncan *post hoc* test. Differences were considered significant at *p* < 0.05. The ANOVA results are reported as an *F*-statistic and its associated degrees of freedom. All graphs were prepared using GraphPad Prism 8.

## Results

### Weight Gain

**FIGURE 1:**
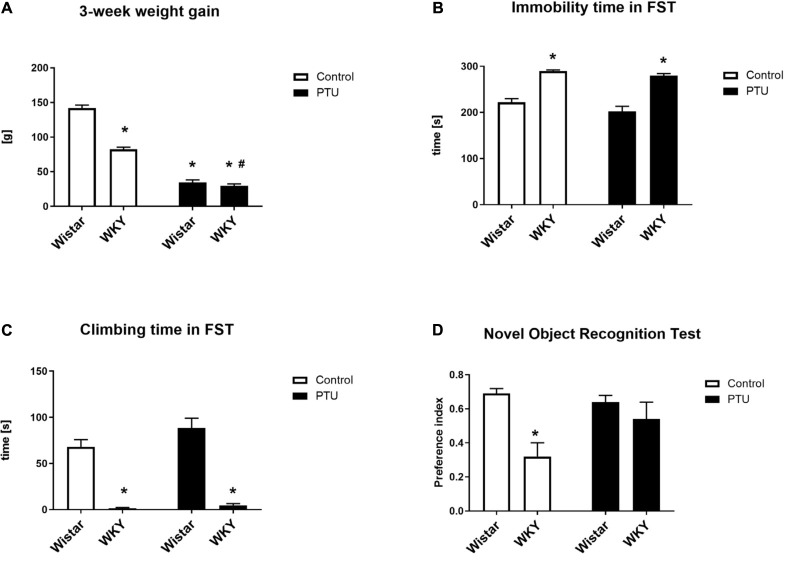
The effects of strain and PTU treatment on weight gain **(A)**, immobility **(B)**, and swimming times **(C)** measured in the forced swim test and on the preference index measured in the novel object recognition test **(D)**. The results are expressed as the mean ± SEM. *p < 0.05 vs. the control group (Wistar rats); ^#^*p* < 0.05 vs. the WKY group. *n* = 9–10.

Factorial ANOVA showed the effects of strain (*F*_1_,_36_ = 81.43, *p* < 0.0001) and PTU (*F*_1_,_36_ = 502.05, *p* < 0.0001) on weight gain over 3 weeks. Weight gain was significantly lower in the WKY rats than in the Wistar rats, and PTU significantly reduced this parameter in both strains (Figure 1A).

### Immobility and the Climbing Time in the Forced Swim Test (Porsolt Test)

The duration of immobility and the climbing time in the forced swim test was measured for 5 min on the second day of the test. In accordance with other data, the WKY rats showed significantly more immobility behavior (strain effect: *F*_1_,_36_ = 98.95, *p* < 0.0001) and less climbing time (strain effect: *F*_1_,_36_ = 128.86, *p* < 0.0001) than the control Wistar rats, i.e., the WKY rats showed depression-like behavior (Figures 1B,C). The administration of PTU had no effect on immobility or climbing time in either strain.

### Short-Term Memory in the Novel Object Recognition (NOR) Test

Short-term memory was investigated with a new object recognition test and is presented as the preference index (Figure 1D). The WKY rats had a significantly lower preference index than the Wistar rats (strain effect: *F*_1_,_35_ = 12.36, *p* = 0.001), but PTU had no effect on short-term memory in either strain.

### Thyroid Hormones and Corticosterone Plasma Concentration

**FIGURE 2:**
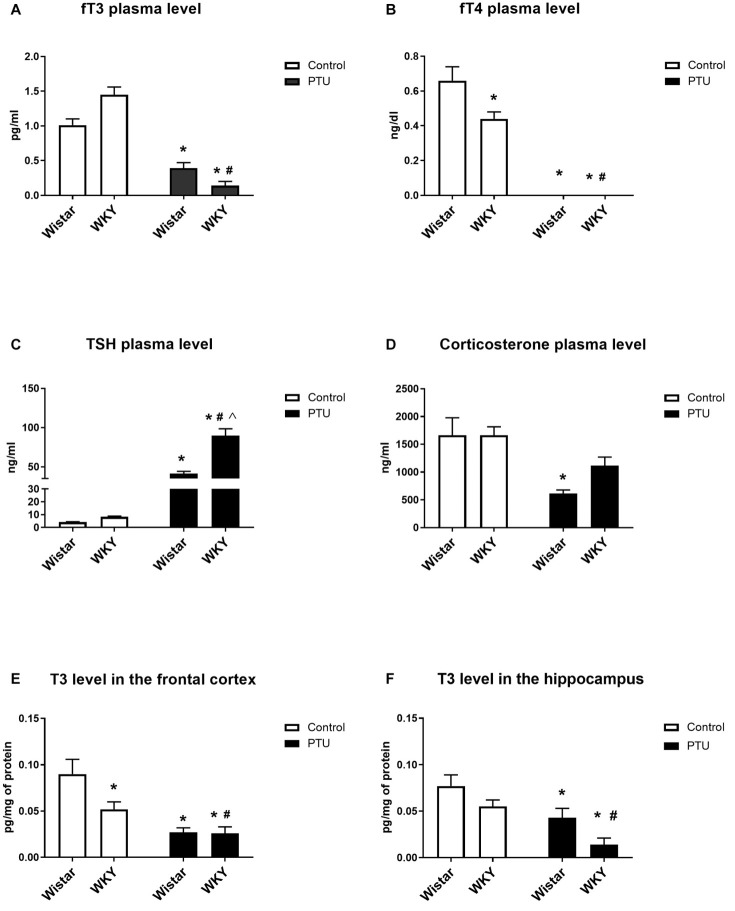
The effects of strain and PTU treatment on fT3 **(A)**, fT4 **(B)**, TSH **(C)**, and corticosterone **(D)** levels measured in the plasma and T3 **(E,F)** levels measured in the frontal cortex and hippocampus. The results are expressed as the mean ± SEM. *p < 0.05 vs. the control group (Wistar rats); ^#^*p* < 0.05 vs. the WKY group; ^Λ^ vs. the Wistar PTU group. *n* = 9–10.

The fT3 concentration was not significantly different between the WKY and Wistar rats, but PTU significantly reduced the levels of this hormone in both strains (treatment effect: *F*_1_,_36_ = 109.58, *p* < 0.0001) (Figure 2A). However, fT4 was significantly lower in the WKY rats than in the Wistar rats (strain effect: *F*_1_,_36_ = 6.12, *p* = 0.018), and PTU (treatment effect: *F*_1_,_36_ = 164.38, *p* < 0.0001) lowered the concentration of this hormone to below the detection limit in both strains (Figure 2B). Plasma TSH levels did not differ significantly between the tested strains, although an upward trend was observed in the WKY rats. PTU significantly increased TSH levels in both strains (treatment effect: *F*_1_,_36_ = 157.39, *p* < 0.0001); however, its effect was significantly stronger in the WKY rats than in the Wistar rats (strain × PTU interaction: *F*_1_,_36_ = 21.86, *p* < 0.0001) (Figure 2C). Corticosterone concentrations were significantly lower in animals receiving PTU than in the control Wistar rats (treatment effect: *F*_1_,_36_ = 17.02, *p* = 0.0002); however, there were no differences in the levels of this hormone between the strains or between the WKY control rats and those receiving PTU (Figure 2D).

### T3 Concentration in the Frontal Cortex and Hippocampus

The T3 levels in the brain were diminished by PTU (treatment effect: *F*_1_,_32_ = 21.06, *p* < 0.0001 for the frontal cortex; *F*_1_,_32_ = 16.09, *p* = 0.0003 for the hippocampus) in both strains and both brain areas (Figures 2E,F). Moreover, *post hoc* analysis indicated that the T3 level in the frontal cortex of the WKY rats was lower than that of the Wistar rats.

### Total Cholesterol, HDL, LDL, and Triglyceride Levels in the Plasma

**FIGURE 3:**
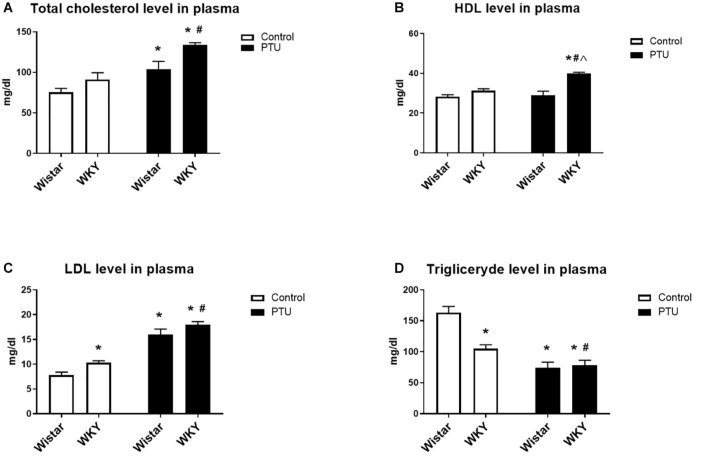
The effects of strain and PTU treatment on the total cholesterol **(A)**, HDL **(B)**, LDL **(C)**, and triglyceride **(D)** levels measured in the plasma. The results are expressed as the mean ± SEM. *p < 0.05 vs. the control group (Wistar rats); ^#^*p* < 0.05 vs. the WKY group; ^Λ^ vs. the Wistar PTU group. *n* = 9–10.

Propylthiouracil significantly increased the levels of total cholesterol in both strains (treatment effect: *F*_1_,_34_ = 25.88, *p* < 0.0001) (Figure 3A). HDL measurements showed the impact of PTU (treatment effect: *F*_1_,_32_ = 13.02, *p* = 0.001) and the strain × PTU interaction (interaction: *F*_1_,_32_ = 9.60, *p* = 0.004). PTU increased the level of HDL in the WKY rats compared with those of the other groups (Figure 3B). The level of LDL was increased in the WKY rats compared with that of the Wistar rats (strain effect: *F*_1_,_33_ = 10.22, *p* = 0.003) and with those of both strains after PTU administration (treatment effect: *F*_1_,_33_ = 131.05, *p* < 0.0001) (Figure 3C). The plasma concentration of triglycerides was diminished in the WKY rats compared with that in the Wistar rats (strain effect: *F*_1_,_33_ = 10.07, *p* = 0.03) and with those in both groups receiving PTU (treatment effect: *F*_1_,_33_ = 46.13, *p* = 0.003) (Figure 3D).

### Gene Expression of Thyroid Receptors (TRα1 and TRβ1) and Retinoid Receptors (RXRα and RXRβ) in the Frontal Cortex and Hippocampus

**FIGURE 4:**
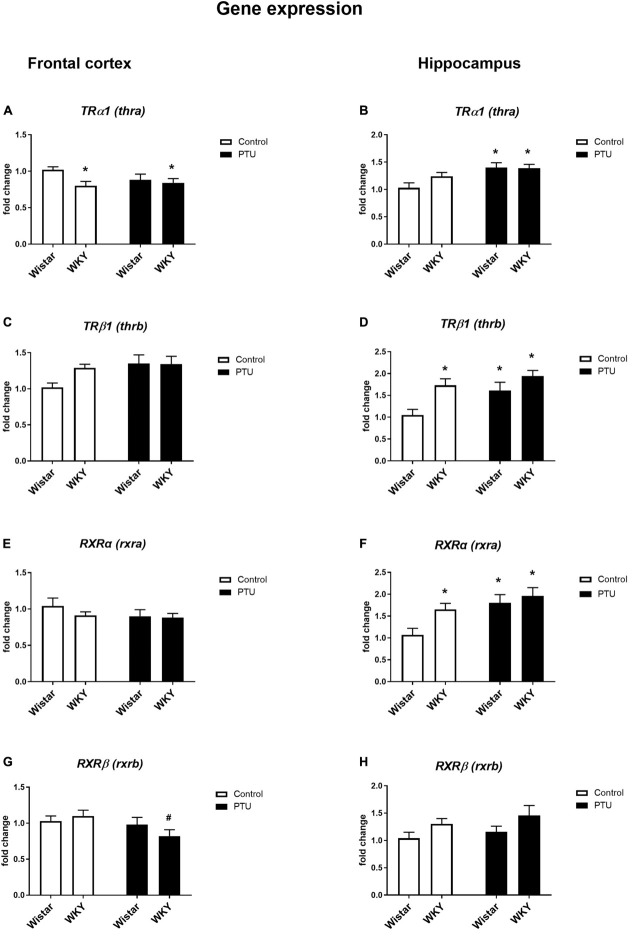
The effects of strain and PTU treatment on the gene expression of the thyroid receptors TRα (*thra*) **(A,B)** and TRβ (*thrb*) **(C,D)** and the retinoid receptors RXRα (*rxra*) **(E,F)**, and RXRβ (*rxrb*) **(G,H)** in the frontal cortex and hippocampus. The results are expressed as the average fold change ± SEM. *p < 0.05 vs. the control group (Wistar rats); ^#^*p* < 0.05 vs. the WKY group. *n* = 6–10.

Significant differences in TRα1 expression in the frontal cortex were observed between the rat strains (strain effect: *F*_1_,_35_ = 4.50, *p* = 0.041), but no effect of PTU was observed. The expression of this receptor was significantly lower in the WKY rats both with and without PTU administration than in the Wistar rats (Figure 4A). There were no differences in TRβ1 or RXRα expression in this brain structure regardless of strain or PTU administration (Figures 4C,E). PTU significantly decreased (treatment effect: *F*_1_,_35_ = 4.58, *p* = 0.039) RXRβ expression in the WKY rats only (Figure 4G).

There was no difference in TRα1 expression in the hippocampus between the rat strains, but PTU administration significantly increased (treatment effect: *F*_1_,_35_ = 11.35, *p* = 0.002) the expression of this receptor subtype in the Wistar rats (Figure 4B). The expression of TRβ1 was significantly higher in the WKY rats than in the Wistar rats (strain effect: *F*_1_,_33_ = 9.88, *p* = 0.004), and PTU (treatment effect: *F*_1_,_33_ = 5.77, *p* = 0.022) increased the expression of this receptor subtype, but this difference reached statistical significance only in the Wistar rats (Figure 4D). Similar to TRβ1, RXRα expression was higher in the WKY rats than in the Wistar rats (strain effect: *F*_1_,_33_ = 4.76, *p* = 0.036), and PTU increased its expression (treatment effect: *F*_1_,_33_ = 9.09, *p* = 0.005) in the Wistar rats only (Figure 4F). There was no effect of PTU on RXRβ expression, and although ANOVA indicated differences between the strains (*F*_1_,_36_ = 4.79, *p* = 0.035), the *post hoc* test showed no significant differences between the Wistar and WKY rats or between those under the control conditions and those that received PTU (Figure 4H).

### Gene Expression of Deiodinase 2 (DIO2) and Deiodinase 3 (DIO3) in the Frontal Cortex and Hippocampus

**FIGURE 5:**
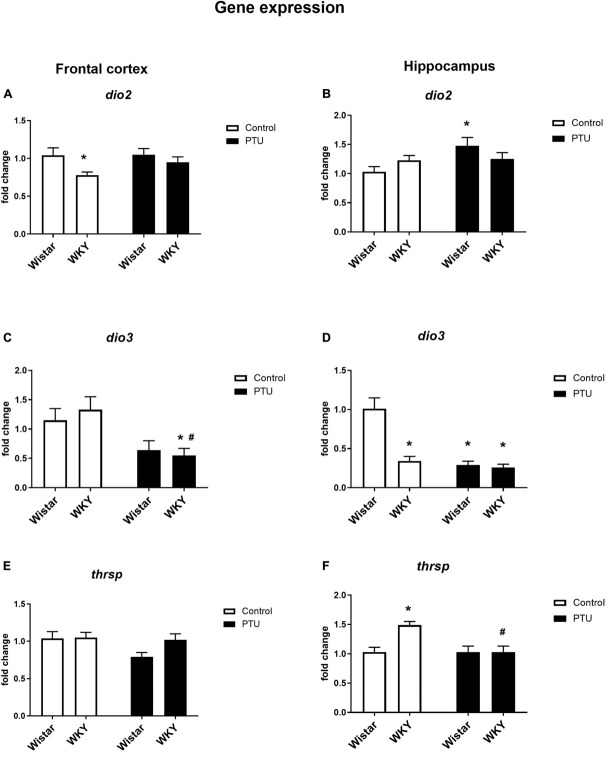
The effects of strain and PTU treatment on the gene expression of deiodinase 2 (*dio2*), **(A,B)** deiodinase 3 (*dio3*) **(C,D)**, and thyroid hormone responsive protein (*thrsp*) **(E,F)** in the frontal cortex and hippocampus. The results are expressed as the average fold change ± SEM. *p < 0.05 vs. the control group (Wistar rats); ^#^*p* < 0.05 vs. the WKY group. *n* = 6–10.

Significant differences between the rat strains (*F*_1_,_34_ = 6.53, *p* = 0.015), but no effect of PTU, was observed in DIO2 expression in the frontal cortex. The expression of DIO2, the enzyme that converts T4 to active T3, was significantly lower in the WKY rats than in the Wistar control rats, whereas in animals receiving PTU, there was no difference in the expression of DIO2 between the rat strains tested (Figure 5A). DIO3 was significantly decreased in the WKY rats by PTU treatment (treatment effect: *F*_1_,_32_ = 11.17, *p* = 0.002), but there were no differences between the strains (Figure 5C).

In the hippocampus, there was no difference in DIO2 expression between the rat strains, and PTU increased this parameter (treatment effect: *F*_1_,_35_ = 4.27, *p* = 0.046) in the Wistar rats only (Figure 5B). In this brain structure, there was a strong reduction in DIO3 expression in the WKY rats compared with that in the Wistar rats and in both strains of animals receiving PTU compared with that in the control (strain effect: *F*_1_,_31_ = 26.28; treatment effect: *F*_1_,_31_ = 34.80, *p* < 0.0001) (Figure 5D).

### Gene Expression of Thyroid Hormone Responsive Protein (THRSP) in the Frontal Cortex and Hippocampus

In the frontal cortex, there were no differences in Thrsp expression between the strains or between the PTU administration and control groups (Figure 5E), but in the hippocampus, the mRNA level of Thrsp was significantly higher in the WKY rats than in the Wistar rats (strain effect: *F*_1_,_34_ = 7.11, *p* = 0.011), and PTU decreased this parameter in the WKY rats (treatment effect: *F*_1_,_34_ = 7.07, *p* = 0.011) (Figure 5F).

### Pyruvate and Lactate Concentrations

**FIGURE 6:**
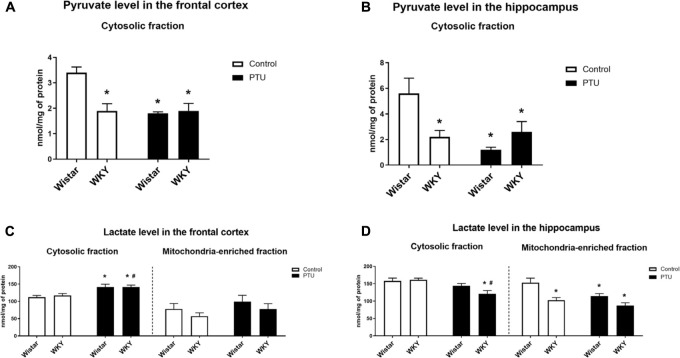
The effects of strain and PTU treatment on the pyruvate level (in cytosolic fraction) **(A,B)** and lactate level (in cytosolic and mitochondria-enriched fraction) **(C,D)** in the frontal cortex and hippocampus. The results are expressed as the mean ± SEM. *p < 0.05 vs. the control group (Wistar rats); ^#^*p* < 0.05 vs. the WKY group. *n* = 7–10.

Pyruvate levels in both the frontal cortex (strain effect: *F*_1_,_28_ = 9.54, *p* = 0.004) and the hippocampus (strain × PTU interaction: *F*_1_,_29_ = 9.98, *p* = 0.004) were significantly lower in the WKY rats than in the control Wistar rats. In the Wistar rats, PTU significantly decreased the pyruvate concentration in both examined brain structures (treatment effect: *F*_1_,_28_ = 12.50, *p* = 0.001 for the frontal cortex; *F*_1_,_29_ = 7.39, *p* = 0.011 for the hippocampus), whereas in the WKY rats, PTU had no effect on the concentration of this glycolysis product (Figures 6A,B).

There was no difference in the level of lactate in the cytosol in either the frontal cortex or hippocampus of the WKY and Wistar rats. PTU (treatment effect: *F*_1_,_32_ = 15.73, *p* = 0.0004) increased lactate levels in the frontal cortex in both the WKY and Wistar rats and decreased (treatment effect: *F*_1_,_33_ = 12.82, *p* = 0.001) lactate concentrations in the hippocampus in the WKY rats only (Figures 6C,D).

The lactate concentration in the mitochondrial fraction of the frontal cortex did not differ between the tested rat strains and was not changed in the animals that received PTU. In contrast to the frontal cortex, in the hippocampus, the lactate level in the mitochondrial fraction was significantly lower in the WKY rats than in the Wistar rats (strain effect: *F*_1_,_29_ = 15.86, *p* = 0.0004), and PTU decreased (treatment effect: *F*_1_,_29_ = 7.66, *p* = 0.010) the lactate level in the Wistar rats (Figures 6C,D).

### Level of Pyruvate Dehydrogenase

**FIGURE 7:**
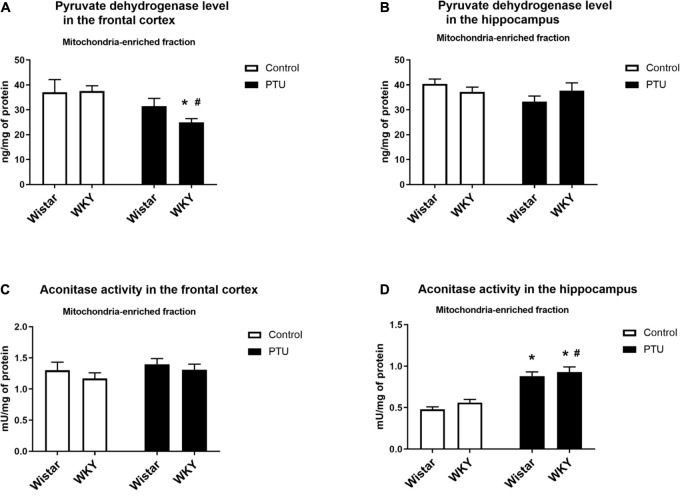
The effects of strain and PTU treatment on the pyruvate dehydrogenase level **(A,B)** and aconitase activity **(C,D)** in the mitochondria-enriched fraction of the frontal cortex and hippocampus. The results are expressed as the mean ± SEM. *p < 0.05 vs. the control group (Wistar rats); ^#^*p* < 0.05 vs. the WKY group. *n* = 7–10.

In the frontal cortex, the level of pyruvate dehydrogenase was significantly lower only in the WKY rats receiving PTU than in the rats under control conditions (treatment effect: *F*_1_,_35_ = 8.13, *p* = 0.007) (Figure 7A), whereas in the hippocampus, the levels of this enzyme were not changed in any treatment group (Figure 7B).

### Aconitase Activity

There were no differences in aconitase activity between the WKY and Wistar rats in either the frontal cortex or the hippocampus. PTU had no effect on the activity of this enzyme in the frontal cortex but significantly enhanced hippocampal aconitase activity in both the Wistar and WKY rats (treatment effect: *F*_1_,_32_ = 77.62, *p* < 0.0001) (Figures 7C,D).

### Levels of Pyruvate Dehydrogenase Kinase Isoform 2 (PDK2) and Pyruvate Dehydrogenase Kinase Isoform 4 (PDK4)

**FIGURE 8:**
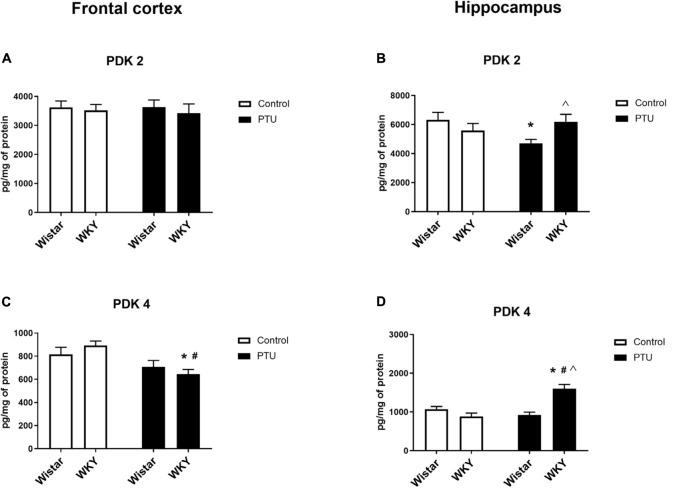
The effects of strain and PTU treatment on PDK2 and PDK4 in the mitochondria-enriched fraction of the frontal cortex **(A,C)** and **(B,D)** hippocampus. The results are expressed as the mean ± SEM. *p < 0.05 vs. the control group (Wistar rats); ^#^*p* < 0.05 vs. the WKY group; ^Λ^ vs. the Wistar PTU group. *n* = 8–9.

In the frontal cortex, the level of PDK2 did not differ among any of the examined groups; however, in the hippocampus, a strain × PTU effect was observed (*F*_1_,_30_ = 6.04, *p* = 0.020). In the Wistar rats, PTU administration significantly diminished the level of PDK2 compared with those in the control Wistar animals and in the WKY rats treated with PTU (Figures 8A,B).

The level of PDK4 in the frontal cortex was decreased by PTU (treatment effect: *F*_1_,_32_ = 13.00, *p* = 0.001) in the WKY rats only (Figure 8C). In the hippocampus, the concentration of PDK4 was upregulated by administration of PTU (treatment effect: *F*_1_,_32_ = 10.39, *p* = 0.003) in the WKY rats. Additionally, the level of PDK4 in PTU-treated animals was increased in the WKY rats compared with that in the Wistar rats (strain × treatment interaction *F*_1_,_32_ = 24.15, *p* < 0.0001) (Figure 8D).

### Protein Expression of Oxidative Phosphorylation (OXPHOS) Complexes

**FIGURE 9:**
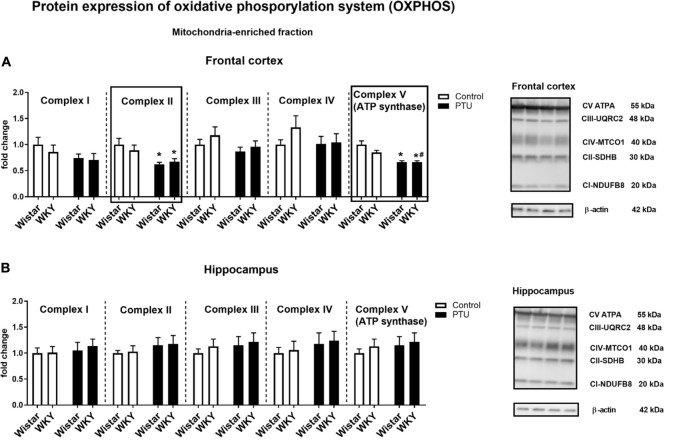
The effects of strain and PTU treatment on the expression of oxidative phosphorylation complexes I–V in the mitochondria-enriched fraction of the frontal cortex **(A)** and hippocampus **(B)**. Representative immunoblots of proteins studied and β-actin in the frontal cortex **(A)** and hippocampus **(B)**. The bands from the left: Wistar, WKY, Wistar PTU, WKY PTU. The results are expressed as the average fold change ± SEM. *p < 0.05 vs. the control group (Wistar rats); ^#^*p* < 0.05 vs. the WKY group. *n* = 8–10.

In the frontal cortex, there was no effect of strain or PTU administration on complexes I, III, and IV, whereas PTU (treatment effect: *F*_1_,_36_ = 12.38, *p* = 0.001) downregulated the levels of complex II in the Wistar and WKY rats compared with those of the untreated Wistar rats. Regarding complex V (ATP synthase), an impact of PTU was observed (treatment effect: *F*_1_,_34_ = 35.90, *p* < 0.0001); diminished levels of this complex were demonstrated in both the Wistar and WKY rats (Figure 9A). There were no changes in the protein levels of respiratory chain complexes in the hippocampus (Figure 9B).

### Mitochondrial Respiration

**FIGURE 10:**
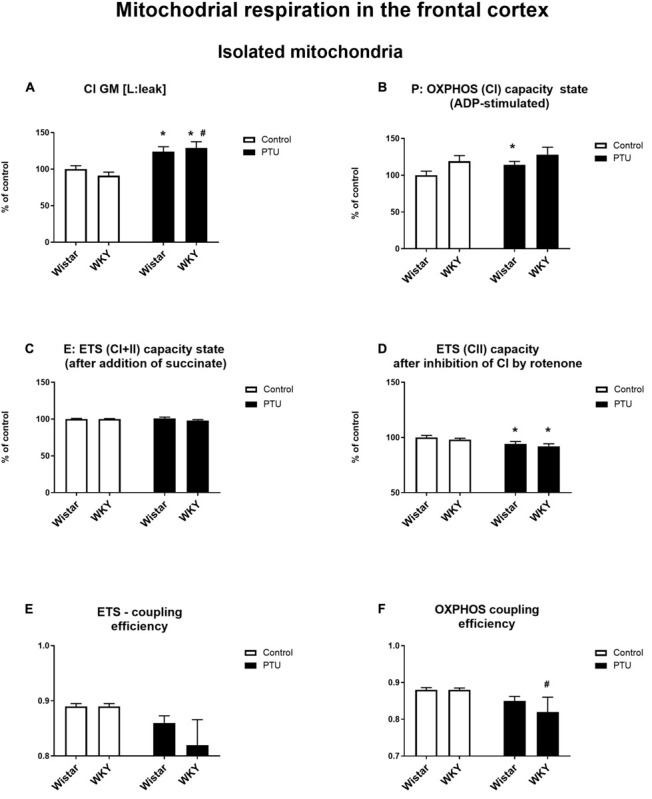
The effects of strain and PTU treatment on the mitochondrial respiration of isolated mitochondria in the frontal cortex: leak state **(A)**, OXHPOS (CI) capacity state **(B)**, ETS (CI + CII) capacity state **(C)**, ETS (CII) capacity state **(D)**, ETS—coupling efficiency state **(E)**, and OXPHOS—coupling efficiency state **(F)**. The results are expressed as the average fold change ± SEM **(A–D)** or as the mean ± SEM. *p < 0.05 vs. the control group (Wistar rats); ^#^*p* < 0.05 vs. the WKY group. *n* = 8–10.

**FIGURE 11:**
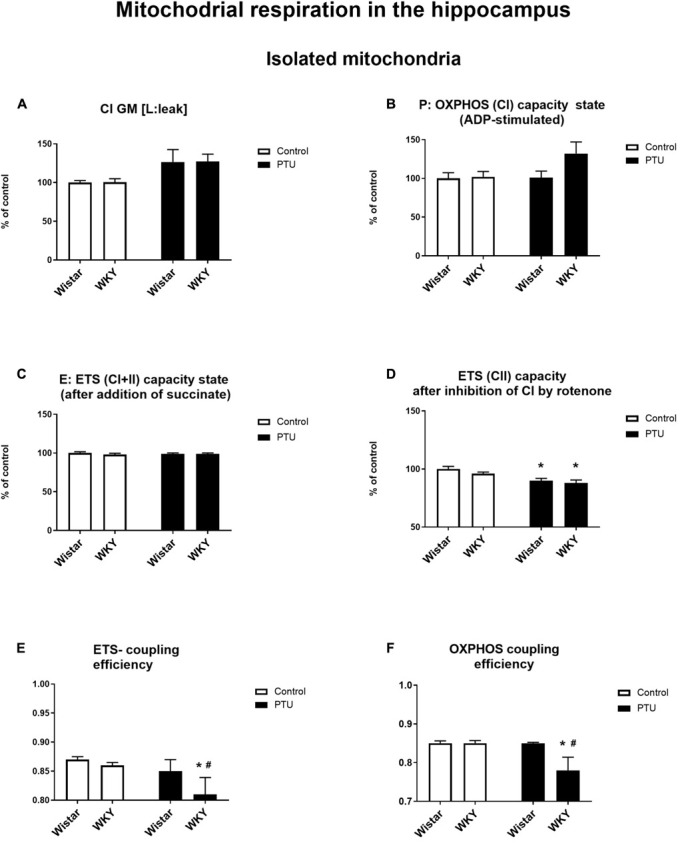
The effects of strain and PTU treatment on the mitochondrial respiration of isolated mitochondria in the hippocampus: leak state **(A)**, OXHPOS (CI) capacity state **(B)**, ETS (CI + CII) capacity state **(C)**, ETS (CII) capacity state **(D)**, ETS—coupling efficiency state **(E)**, and OXPHOS—coupling efficiency state **(F)**. The results are expressed as the average fold change ± SEM **(A–D)** or as the mean ± SEM. *p < 0.05 vs. the control group (Wistar rats); ^#^*p* < 0.05 vs. the WKY group. *n* = 8–10.

High-resolution respirometry (HRR) was used to measure mitochondrial respiration in both selected brain structures. In the frontal cortex, PTU (treatment effect: *F*_1_,_33_ = 15.72, *p* = 0.0004) increased leak state [L: LEAK (CI)] respiration in the presence of complex I substrates (malate and glutamate) in both strains (Figure 10A). The P: OXPHOS (CI) capacity (measured after the addition of ADP) in the frontal cortex was significantly enhanced in the Wistar rats treated with PTU compared to that in the control group (treatment effect: *F*_1_,_34_ = 4.29, *p* = 0.046) (Figure 10B). In the hippocampus, there were no differences in these two respiratory states among the groups (Figures 11A,B). Furthermore, no effect of strain or PTU was observed in the E: ETS (CI + II) capacity after the addition of succinate in either the frontal cortex or the hippocampus (Figures 10C, 11C). However, the E: ETS (CII) capacity (measured after the inhibition of CI by rotenone) was decreased in the Wistar and WKY rats treated with PTU (treatment effect: *F*_1_,_33_ = 9.19, *p* = 0.005 for the frontal cortex; *F*_1_,_35_ = 20.55, *p* < 0.0001 for the hippocampus) compared with those in the control Wistar rats in both brain areas (Figures 10D, 11D).

In addition, a mitochondrial respiration assay showed that PTU decreased ETS and OXPHOS coupling efficiency in the brain (ETS: *F*_1_,_35_ = 5.21 for the hippocampus, *p* = 0.0286; OXPHOS: treatment effect: *F*_1_,_36_ = 5.16 for the frontal cortex, *p* = 0.029; *F*_1_,_35_ = 5.17 for the hippocampus, *p* = 0.029), while the *post hoc* tests showed a significant decrease in ETS and OXPHOS coupling efficiency only in the hippocampus of the WKY rats after PTU administration and in OXPHOS coupling efficiency in the frontal cortex in the same group of animals (Figures 10F, 11E,F). No significant changes in ETS coupling efficiency were observed in the frontal cortex (Figure 10E).

### Protein Expression of Mitofusin 2, UCP4, VDAC1, and HK1

There were no changes in the level of mitofusin 2 between the WKY and Wistar rats in either the frontal cortex or the hippocampus. PTU also had no effect on the expression of this protein. UCP4 expression was diminished in the WKY rats treated with PTU compared with its expression in the Wistar rats (treatment effect: *F*_1_,_31_ = 5.35, *p* = 0.028) in the frontal cortex only. In the hippocampus, the level of UCP4 did not differ among any of the examined groups. The level of VDAC1 in either brain structure did not differ between the tested rat strains and was not changed in the animals that received PTU. HK1 expression in the hippocampus was increased in the WKY animals treated with PTU compared with its expression in all other groups (strain × PTU interaction: *F*_1_,_33_ = 4.71, *p* = 0.037) (Table 1).

**TABLE 1:** The effects of strain and PTU treatment on the level of Mitofusin 2, UCP4, VDAC1, and HK1 in the mitochondria-enriched fraction of frontal cortex and hippocampus.

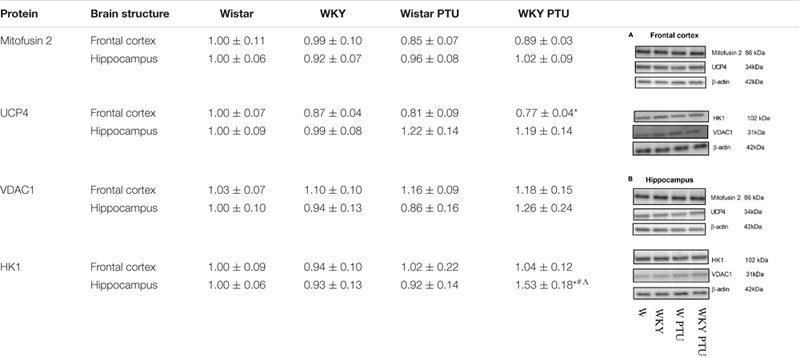

*Representative immunoblots of proteins studied and β-actin in the frontal cortex **(A)** and hippocampus **(B)**. The bands from the left: Wistar, WKY, Wistar PTU, WKY PTU. The results are presented as fold change and expressed as the means ± the SEMs.^∗^p < 0.05 vs. control group (Wistar rats); ^#^p < 0.05 vs. the WKY group; ^Λ^ vs. Wistar PTU group. n = 8–10.*

## Discussion

In the present study, we found that there were changes in glucose metabolism in the frontal cortex and/or hippocampus in an animal model of depression (WKY rats) and in a model of the co-occurrence of depression and hypothyroidism (WKY rats receiving PTU) compared to the control Wistar rats. In addition, the decreased metabolism in the frontal cortex in depression may result from decreases in the levels of T3 and TRα1 and in the expression of DIO2, an enzyme that catalyzes the conversion of T4 to the active T3 hormone.

As in previous studies, in the forced swim test, we observed a longer duration of immobility and a shorter climbing time in the WKY rats than in the control Wistar animals (Aleksandrova et al., 2019). Additionally, in agreement with previous data, the WKY rats showed short-term memory impairment and reduced weight gain. The administration of PTU had no effect on immobility or climbing time but attenuated the memory impairment of the WKY rats and intensified the decrease in weight gain. It is difficult to explain that in contrast to our predictions, the administration of PTU did not lead to prolonged immobility time in the Porsolt test in either the Wistar or WKY rats. Although behavioral tests, including the Porsolt test, have been widely used to screen potential antidepressant drug effects, whether they can assess depression in rodents remains a matter of debate (Yu et al., 2015). It is still unknown which of the numerous changes observed in the human brain or in animal models of depression (e.g., changes in neurotransmitters, hormones, cytokines, intracellular signal transduction, transcription factors, metabolic processes, etc.) are crucial in the development of depression, and behavioral tests based primarily on changes in noradrenergic and/or serotonergic neurotransmission may not necessarily reflect the key targets in depression. It is also unresolved why in many animal models of depression based on stress procedures, not all animals develop depression-like behavioral changes despite the use of identical stressors, whereas the biochemical changes are most often similar (Kolasa et al., 2014). Since depression is a highly heterogeneous disorder with diverse pathogenic origins, the lack of changes in depression-like behavior does not exclude the participation of other factors, including hypothyroidism, that do not affect the time of immobility but affect other processes (e.g., cause metabolic disturbances) in the pathogenesis of this disorder. For thyroid hormones, data on their effects on depression-like and anxiety-like behaviors are very ambiguous because both hypothyroidism and hyperthyroidism can induce such behaviors. Additionally, concerning hypothyroidism, some studies found an increase in depression-like behavior in rats, but others found decreased depression-like behavior (Yu et al., 2015). Furthermore, hypothyroidism in humans does not necessarily lead to depression but increases the risk of this disease, and the administration of a high dose of T4 to patients with drug-resistant depression produces a therapeutic effect, probably by normalizing any significant changes that have occurred in depression. We also verified the hypothyroidism model by determining the plasma lipid levels. Total cholesterol and low-density lipoprotein cholesterol were significantly increased under hypothyroidism conditions in both rat strains; however, high-density lipoprotein cholesterol was elevated only in the WKY rats. In agreement with previous data, the lowered thyroid hormone levels in rats of both strains decreased triglyceride levels in the blood, which contrasts with the effect observed in humans (Popović et al., 1998).

The results obtained in the current research confirmed and extended the data showing the presence of hypothalamic–pituitary–thyroid (HPT) axis disturbance in WKY rats and indicated the possible causes of the weaker action of these hormones in the brain compared to their action in peripheral tissue. In the blood, both TSH and fT3 levels tended to be higher in the WKY rats than in control animals, and although these changes did not reach statistical significance, they suggested a weakening of the inhibitory effect of T3 on TSH secretion (i.e., the attenuation of the feedback mechanism regulating HPT axis activity). As in the current research, Redei et al. (2001) found higher levels of both TSH and T3 in the WKY rats than in the Wistar rats. In a previous study, the level of total T3 was measured, whereas we studied the free fraction of this hormone, which not only confirmed previous data but also excluded the effects of potential differences in thyroid hormone-binding protein levels on the active fraction of this hormone in the WKY and Wistar rats. The total T4 hormone levels were slightly but not significantly lower in WKY rats than in Wistar rats in the Redei study (Redei et al., 2001), while we found that the level of the free form of this hormone was significantly lower in the WKY rats than in the Wistar rats.

Although the blood concentration of fT3 tended to be higher in the WKY rats than in the Wistar rats, the level of this hormone in the frontal cortex of the WKY rats was lower, and its level in the hippocampus was approximately 30% lower, although this difference did not reach statistical significance. The fact that thyroid hormone levels in the blood do not correlate with their concentrations in the brain is known, and it follows that the T3 content in the brain depends not only on its synthesis in the thyroid gland but also, to a large extent, on the expression of transporters and deiodinase enzymes in particular brain structures. It is estimated that only approximately 20% of brain T3 is derived from the blood, and the majority is formed in glial cells from thyroxine under the action of DIO2 (Morte and Bernal, 2014). Since the expression of DIO2 in the frontal cortex was lower in the WKY rats than in the Wistar rats, this could be one of the reasons for the lower T3 concentration we observed in the frontal cortex of the WKY rats. However, it should be noted that many factors affect the levels of thyroid hormones and deiodinases in the brain, and additionally, the roles of iodothyronine derivatives other than T3 and T4 are poorly known (Pinna et al., 2002); therefore, it is not possible to predict the actions of these hormones in the brain based only on their blood or tissue concentrations and on the expression of deiodinases and transporters.

The predominant mechanism for the biological action of TH involves the regulation of gene transcription through nuclear TH receptors. The effects of TH depend mainly on the expression of specific isoforms of TH receptors and their dimerization with RXR. The decreased expression of the TRα1 isoform in the frontal cortex of the WKY rats suggested weaker TH activity in this brain structure because this receptor isoform is widely distributed and is the predominant TH receptor in the adult brain. TRα1 presumably mediates most of the effects of TH, and some studies show that mutation of this receptor increases depressive and anxiety behaviors, evokes memory impairment, and reduces glucose utilization in the brain (Cheng, 2005; Vallortigara et al., 2009; Pilhatsch et al., 2010; Richard et al., 2017). Moreover, in a chronic mild stress model of depression, a decrease in TRα1 mRNA expression in the brain and its reversal by imipramine suggest that this receptor may play a role in stress-induced depressive behavior and in antidepressant action (Stein et al., 2009).

In contrast to the frontal cortex of the WKY rats, in which changes in the levels of DIO2 and TRα1 receptor suggest that a decrease in thyroid hormone action, in the hippocampus, the severe reduction in the expression of DIO3, the T3- and T4-metabolizing enzyme, and the increase in the expression of TRβ1 and RXRα indicate the enhancement of thyroid hormone function. However, the comparison of selected metabolic markers in these brain structures in the WKY and Wistar rats indicated that a decrease in metabolism occurred not only in the frontal cortex but also in the hippocampus.

A decrease in glycolysis was evidenced by the finding that the level of pyruvate, the end product of glycolysis, was lower in both brain structures studied in the WKY rats than in the Wistar rats; additionally, the level of pyruvate was lower in both strains with hypothyroidism than in the strains under control conditions. In the frontal cortex in the hypothyroidism models but not in the depression model, reduced levels of pyruvate may be caused by the increased conversion of pyruvate to lactate. However, in the WKY rats receiving PTU, a simultaneous decrease in pyruvate dehydrogenase levels and pyruvate levels suggested the weakening of the Krebs cycle by limiting both its substrate and the enzyme linking glycolysis with the Krebs cycle. Moreover, in addition to a reduction in pyruvate dehydrogenase levels, a decrease in pyruvate dehydrogenase kinase 4, the enzyme that inhibits pyruvate dehydrogenase, was observed only in the model of depression with hypothyroidism. These changes indicate that lower levels of pyruvate dehydrogenase in the frontal cortex can be partially compensated by an increase in its activity. In contrast to the cortex, in the hippocampus, there was no decrease in the level of pyruvate dehydrogenase; however, the increased PDK4 level suggested that the activity of this enzyme could have been decreased. In the hippocampus, the decreased pyruvate levels were accompanied by reduced lactate concentrations in the mitochondrial fractions of all treated animal groups compared to that of the control Wistar rats; moreover, in the model of depression with hypothyroidism, lactate concentrations in the hippocampus were also reduced in the cytosolic fraction, which was even more clear than in the case of the frontal cortex, indicating a decrease in glycolysis efficiency. Furthermore, a significant decrease in the level of lactate, which can act as a gliotransmitter in addition to being an energy substrate mainly for neurons, may lead to a reduction in norepinephrine release and disturbances in long-term memory (Suzuki et al., 2011; Tang et al., 2014). Some existing data indicate that hypothyroidism induces the detachment of hexokinase 1 (HK1) from the outer mitochondrial membrane by lowering the expression of voltage-dependent anion channel 1 (VDAC1), and this action may be an important cause of the disrupted coordination between glycolysis and oxidative phosphorylation (Regenold et al., 2012). In our study, the levels of VDAC1 in the mitochondrial fraction in both brain structures examined did not change, and the HK1 concentration did not decrease or even increased in the hippocampus of the WKY rats treated with PTU; therefore, we excluded the participation of this mechanism in the models we examined.

In the frontal cortex, the decreased expression of complex II and complex V in rats with hypothyroidism suggested that in this brain structure, not only glycolysis but also oxidative phosphorylation could be reduced. However, since no differences in the expression of these enzymes were observed between the rat strains, only slight downward trends in these levels were observed in the WKY rats, and because PTU decreased the levels of these enzymes in the WKY and Wistar rats to a similar extent, the changes in complexes II and V may be associated with hypothyroidism but not with differences between the tested rat strains. Additionally, functional assays of mitochondrial respiration showed the impact of PTU, but not of strain, on the leak and OXPHOS states. The intensification of the leak state in animals with hypothyroidism could indicate the uncoupling of oxidative phosphorylation from ATP synthesis in these animals. In the leak state, when ATP synthase is not active, oxygen consumption is mainly used to compensate for proton leakage. Thus, the increase in oxygen consumption in the leak state in animals receiving PTU suggested that in the rats with hypothyroidism, the inner mitochondrial membrane was more permeable to protons, and consequently, oxidative phosphorylation may have become uncoupled from ATP synthesis. This change is difficult to explain because T3 is known to activate thermogenesis by uncoupling electron transport from ATP synthesis in brown adipose tissue (BAT) mitochondria, whereas the level of this hormone in the frontal cortex in rats treated with PTU was decreased. However, the action of thyroid hormones in BAT is relatively well studied, and it is known that T3 increases fatty acid oxidation and mitochondrial respiration and also affects the processes of mitophagy and biogenesis. However, little is known about the effect of thyroid hormones on mitochondrial function in adult brain cells. Neural tissue shows a very high respiratory activity that may exceed several times those of other metabolically active peripheral tissues, and the action of thyroid hormones on metabolic activity, the process of mitochondrial disintegration (fission and mitophagy), and the process of mitochondrial formation (biogenesis and fusion) in brain cells is poorly studied. Thus, differences in the action of thyroid hormones on the mitochondrial uncoupling states of the brain and peripheral tissues may result from many factors; for example, thyroid hormone deficiency can cause differences in the expression and function of uncoupling proteins and low contents of antioxidant enzymes in the brain as well as affect the mitochondrial membrane potential of intracellular signaling pathways. Additionally, we found that the uncoupling of oxidative phosphorylation from ATP synthesis did not result from the overexpression of uncoupling protein 4 (UCP4). The main UCP isoforms expressed in the brain are UCP2, UCP4, and UCP5, and it has been found that overexpression of UCP4 and UCP5, but not UCP2, reduces mitochondrial membrane potential, decreases ATP production and reduces the release of H_2_O_2_ by astrocytes (Lambert et al., 2014). Moreover, in some cellular models, UCP4 has been shown to increase mitochondrial complex II activity and glucose uptake and shift ATP production from mitochondrial respiration to glycolysis (Ramsden et al., 2012). The reduction in UCP4 expression in the frontal cortex in the model of the co-occurrence of hypothyroidism and depression seems to be an adaptive mechanism caused by the weakening of oxidative phosphorylation in this brain structure. The adverse effects of decreased thyroid hormone levels on mitochondrial respiration were also evidenced by reductions in the ETS (CII) state in the frontal cortex and hippocampus. Moreover, PTU decreased the ETS coupling efficiency and OXPHOS coupling efficiency. Since a significant decrease in these parameters after PTU treatment was observed only in the WKY rats, it seems that although decreased coupling efficiency is mainly an effect of hypothyroidism, in the presence of hypothyroidism and depression, this decrease was more pronounced. Summarizing the metabolic effects in the brain caused by the decreased synthesis of thyroid hormones, it seems that, similar to peripheral tissues, oxidative phosphorylation is reduced, since we observed decreased expression of complex II and V in the frontal cortex and decreased ETS (CII) capacity in both brain structures examined. Regarding the earlier stages of metabolism, the process of glycolysis was also decreased in both brain structures, but the Krebs cycle was only reduced in the frontal cortex, while in the hippocampus, it was intensified. This suggests that in the hippocampus, in contrast to the frontal cortex but similar to the case of changes in the expression of T3 receptors, metabolic compensatory mechanisms were also activated.

It is known that the proper function of mitochondria depends on maintaining a balance between fusion and fission. In the case of mitochondrial dysfunction, the intensification of fusion increases the production of ATP and is considered a neuroprotective mechanism (Martorell-Riera et al., 2014); therefore, the expression of the main fusion-mediating protein, mitofusin 2, was determined. We did not observe changes in the expression of mitofusin 2 in any of the animal groups studied, which suggests that there are no changes in the mitochondrial fusion process in the model of depression applied in this study or in animals with hypothyroidism.

An interesting result of the current research was that the gene encoding thyroid hormone-responsive protein (THRSP) was more highly expressed in the hippocampus of the WKY rats than in the hippocampus of the Wistar rats. THRSP is one of the few genes in the adult brain that are sensitive to thyroid hormones, and its activation by thyroid hormones is considered to cause the cytotoxic effect of thyroid hormones observed in primary neuronal cell cultures (Haas et al., 2005). However, most studies indicate that thyroid hormones have a neuroprotective effect and suggest that the hippocampus is especially sensitive to their action. For example, hypothyroidism is associated with decreases in the number of neurons in CA1, CA3, and the dentate gyrus; in the volume of the granular cell layer; in hippocampus-dependent cognitive function; in cholinergic system activity; and in adult hippocampal neurogenesis (Madeira et al., 1992; Alzoubi et al., 2009; Remaud et al., 2014; Wang et al., 2015). Thus, increased THRSP expression in the hippocampus of WKY rats could be connected with the disturbances in synaptic plasticity demonstrated in this rat strain (Aleksandrova et al., 2019). Animals with increased THRSP concentrations in the striatum exhibited inattention behavior, but the function of this protein in the hippocampus has not yet been studied (Custodio et al., 2018). However, it should be taken into account that both impaired synaptic plasticity, which is considered crucial in the pathogenesis of depression, and hypothyroidism-induced disturbances in mitochondrial function can be caused by many factors, e.g., elevated glucocorticoid levels or a high-fat diet (HFD). HFD consumption is known to cause systemic and central inflammation, mitochondrial dysfunction, and excessive ROS production, which can lead to disturbances in synaptic plasticity and consequently increase the risk of developing mental and neurodegenerative disease (Crispino et al., 2020).

Our study has some limitations. The first limitation is that only two behavioral tests were performed: the forced swim test and the novel object recognition test. Perhaps for this reason, we did not find an impact of PTU on depression-like behavior or on recognition memory in the WKY rats. However, greater numbers of behavioral tests, especially the sucrose preference test, increase the probability of the tests affecting metabolic markers in the brain, which were the main parameters measured in this research. Additionally, the substantial decrease in weight gain in the WKY rats and in both strains after PTU administration can be interpreted as a symptom of depression; however, its impact on the behavioral tests and the metabolic parameters cannot be excluded. Another significant limitation of the current research is the use of only male rats, which did not allow us to determine the sex-related differences in the parameters tested. Finally, the physiological importance of the reduced level of the gliotransmitter lactate and the increased expression of cytotoxic THRSP in the hippocampus in the model of depression used in this study should be determined in further studies.

In summary, the obtained results suggested that in both the depression and hypothyroidism models, a reduction in glycolysis and in the connection between glycolysis and the Krebs cycle occurred, while the weakening of oxidative phosphorylation was mainly due to a lower level of thyroid hormones. However, the co-occurrence of hypothyroidism and depression led to changes in the presence or abundance of some metabolic markers, such as decreased pyruvate dehydrogenase levels in the frontal cortex, decreased lactate levels in the cytosolic fraction of the hippocampus, increased PDK4 expression in the hippocampus, and decreased ETS and OXPHOS coupling efficiency. We have also found that the changes observed in the studied models depend on which brain structure is evaluated. Alterations in markers that determine the effect of thyroid hormones in the frontal cortex clearly indicated that in the studied model of depression, their function could be weakened (as shown by the decreases in T3, TRα1, and DIO2), while in the hippocampus, only a downward trend in the T3 level was observed, while the changes in TRβ1, RXRα, and DIO3 suggested the induction of compensatory mechanisms for enhancing T3 action. Similarly, in the rats receiving PTU, the expression of T3 receptors was increased in the hippocampus but not in the frontal cortex, probably as a mechanism to compensate for the lower concentration of this hormone. These changes are consistent with many results indicating that, under the influence of unfavorable factors, protective mechanisms are induced to a greater extent in the hippocampus than in the frontal cortex (Kucharczyk et al., 2018). Comparing the metabolic changes, a greater reduction in the glycolysis process was observed in the hippocampus than in the frontal cortex; in contrast, the Krebs cycle was weakened only in the frontal cortex. Additionally, changes in oxidative phosphorylation were present mainly in the frontal cortex, but these disturbances were related to a deficiency of thyroid hormones rather than depression. Thus, the weakening of the glycolysis process occurred in both brain structures examined, while changes in the later stages of metabolism prevailed in the frontal cortex.

## Data Availability Statement

The data supporting the conclusions of this article will be made available by the authors, without undue reservation, to any qualified researcher. Requests to access the datasets should be directed to KG, glombik@if-pan.krakow.pl.

## Ethics Statement

The animal study was reviewed and approved by Local Ethics Committee in Kraków, Poland (permission no. 46/2018 of 01.02.2018).

## Author Contributions

KG and BB: conceptualization, writing—original draft, and writing—review. KG, JD, and BB: formal analysis. KG, JD, and AK: investigation. KG: methodology and visualization. All authors contributed to the article and approved the submitted version.

## Conflict of Interest

The authors declare that the research was conducted in the absence of any commercial or financial relationships that could be construed as a potential conflict of interest.
